# Exploring the Antimicrobial Potential and Biofilm Inhibitory Properties of Hemocyanin from *Hemifusus pugilinus* (Born, 1778)

**DOI:** 10.3390/ijms241411494

**Published:** 2023-07-15

**Authors:** Sivakamavalli Jeyachandran, Hethesh Chellapandian, Kiyun Park, Ihn-Sil Kwak

**Affiliations:** 1 Lab in Biotechnology & Biosignal Transduction, Department of Orthodontics, Saveetha Dental College & Hospitals, Chennai 600077, Tamil Nadu, India; 2 Fisheries Science Institute, Chonnam National University, 50 Daehak-ro, Yeosu 59626, Republic of Korea; ecoblue@hotmail.com; 3 Department of Ocean Integrated Science, Chonnam National University, Yeosu 59626, Republic of Korea

**Keywords:** *Hemifusus pugilinus* (Born, 1778), hemocyanin, agglutination, antifungal activity, antibiofilm, antimicrobial

## Abstract

The seafood industry plays a huge role in the blue economy, exploiting the advantage of the enriched protein content of marine organisms such as shrimps and molluscs, which are cultured in aquafarms. Diseases greatly affect these aquatic organisms in culture and, hence, there is need to study, in detail, their innate immune mechanisms. Hemocyanin is a non-specific innate defense molecule present in the blood cells of several invertebrates, especially molluscs, arthropods, and annelids. It is concerned with oxygen transport, blood clotting, and immune enhancement. In the present study, this macromolecular metalloprotein was isolated from the hemolymph of the marine snail *Hemifusus pugilinus* (Born, 1778) using Sephadex G-100 gel filtration column chromatography. It occurred as a single band (MW 80 kDa) on SDS-PAGE. High-performance liquid chromatography (HPLC) of the purified hemocyanin showed a single peak with a retention time of 4.3 min. The secondary structure and stability of the protein were detected using circular dichroism (CD), and the spectra demonstrated negative ellipticity bands close to 208 nm and 225 nm, indicating β-sheets. Further exploration of the purified hemocyanin revealed remarkable antimicrobial and antibiofilm activities against Gram-positive (*Enterococcus faecalis* and *Staphylococcus aureus*) and Gram-negative bacteria (*Pseudomonas aeruginosa* and *Proteus vulgaris*) at a concentration of 1–5 μg/mL. Spectrophotometric and in situ microscopic analyses (CLSM) unveiled the potential of the purified hemocyanin to inhibit biofilm formation in these bacteria with a minimal inhibitory concentration of 40 μg/mL. Furthermore, *H. pugilinus* hemocyanin (10 μg/mL concentration) displayed antifungal activity against *Aspergillus niger*. The purified hemocyanin was also assessed for cytotoxicity against human cancer cells using cell viability assays. Altogether, the present study shows that molluscan hemocyanin is a potential antimicrobial, antibiofilm, antifungal, anticancer, and immunomodulatory agent, with great scope for application in the enhancement of the immune system of molluscs, thereby facilitating their aquaculture.

## 1. Introduction

Generally, molluscs are deprived of adaptive immunity so they depend on innate immunity, facilitated by cellular and humoral immune reactions, to combat microbial infections [1]. Cellular immunity mainly involves phagocytosis by hemocytes, but humoral immunity requires the release of antimicrobial factors that exterminate pathogens [2]. In the molluscan humoral system, hemocyanin, a respiratory protein, is also capable of antibacterial action in the host’s immunity upon the entry of pathogens [3,4,5]. The antibacterial and antibiofilm activities of hemocyanin have been reported in various investigations on invertebrates, including molluscs [6,7,8,9] and arthropods [6,10,11,12,13].

Hemocyanin is a macromolecular metalloprotein structurally related to prophenoloxidase (proPO) [14,15]. proPO is a crucial immune molecule present in many invertebrate species that has developed strong pathogenic responses inside hemocytes [15] or after activation via the secretion of proteases by pathogens [16]. Phenoloxidase participates in the melanin production cascade and in the formation of several proteases, protease inhibitors, pattern recognition receptors and regulatory proteins. Several steps are involved in the cascade to produce the final product quinone, which is a kind of reactive oxygen species (ROS) [17]. The use of ROS is a potential approach in invertebrates to counteract bacterial infection [16]. Furthermore, several studies have demonstrated abalone hemocyanin’s mediating role as a precursor for antimicrobial peptides (AMP) [9,18]. AMPs, such as haliotisin, abhisin and defensins, are produced due to abalone hemocyanin. These AMPs have demonstrated broad-spectrum antimicrobial properties against both Gram-negative and Gram-positive bacteria [18,19,20], which they mediate by binding to DNA and RNA, damaging bacterial membranes and directly inhibiting bacterial cell growth. A strong bacteriostatic effect with these AMPs suggests that one AMP could have several antibacterial mechanisms. These data provide an explanation for the diverse antibacterial activity of AMPs produced from abalone hemocyanin, which may be helpful to combat antibiotic-resistant bacteria. In addition, it also possesses prominent antimicrobial compounds [21,22,23,24]. These include depsipeptide, peptide, sterols, terpenes, sesquiterpene, polypropionate, macrolides, nitrogenous compounds, fatty acid derivatives and prostaglandins, miscellaneous compounds, alkaloids and sterols, where all these compounds represent several prominent activities [25,26,27]. In more recent times, investigations on *Crassostrea gigas* [28], *Apostichopus japonicas* [29] and *Biomphalaria glabrata* [30] have unveiled antibacterial and antiviral potential. In this respect, investigations have been conducted on the Indian marine gastropod mollusc *Hemifusus pugilinus* only sparingly. The function of hemocyanin in *H. pugilinus* has not yet been studied, and is the ultimate aim of this investigation. In this study, *H. pugilinus* hemocyanin was purified and assessed for antimicrobial, antibiofilm and cytotoxic properties, and the results substantiate its immune responses against pathogens, which could be useful for healthy culture management, disease prevention and disease control in gastropod molluscs.

## 2. Results and Discussion

Molluscs have developed an enormously defined innate immune system to protect against invasive pathogens through immune recognition, signal transduction and various antimicrobial responses. Numerous studies have been executed on the innate immunity of molluscs, including studies on immune proteins, bioactive compounds, drugs and food additives, because of their huge economic significance. Owing to their importance in the aqua- and pharmaceutical industries, frequent disease outbreaks during the cultivation of molluscs are gaining attention in this research area [31]. A broad range of molecules were isolated from marine invertebrates, including bivalves [32,33,34]. The hemolymph of numerous molluscan species, such as sea hares, oysters, sea slugs and mussels, demonstrated remarkable antimicrobial activity against pathogenic microbes [27,35,36,37]. The present study focused on the purification, for the first time, of the hemocyanin of *H. pugilinus* (Mollusca, Gastropoda) and the analysis of its functional properties using microscopic and spectrophotometric techniques.

**Figure 1:**
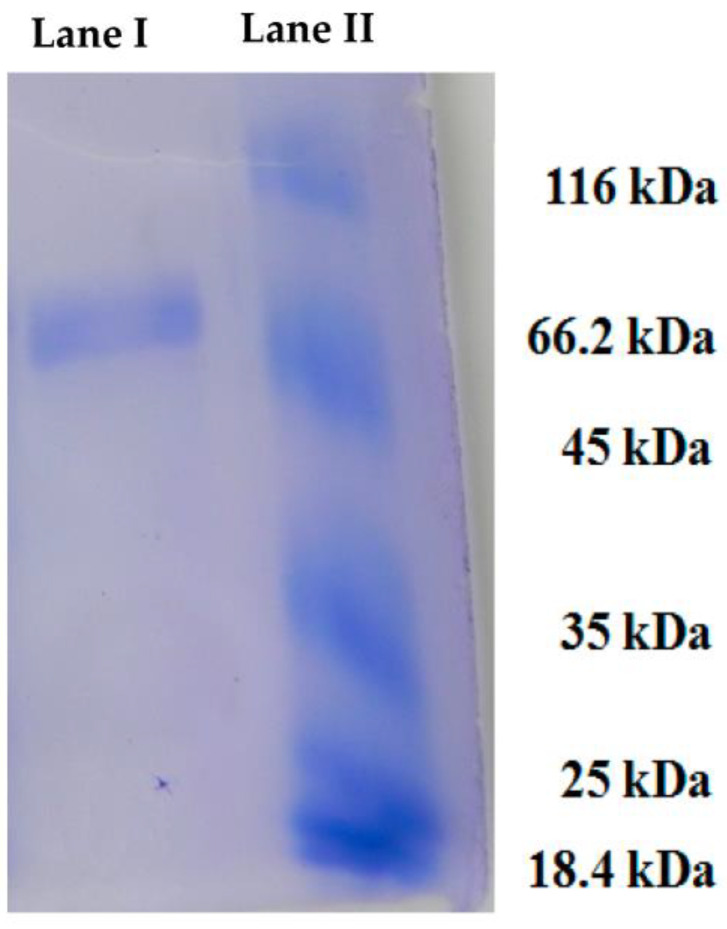
Purified hemocyanin from the hemolymph of *H. pugilinus* on SDS-PAGE. Lane I, protein molecular marker, mw appropriately 80 kDa; Lane II, purified hemocyanin.

**Figure 2:**
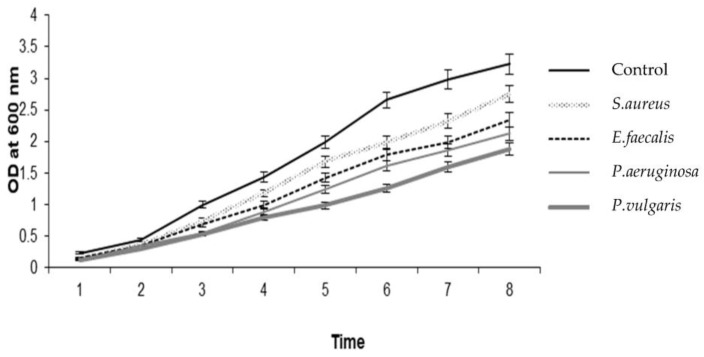

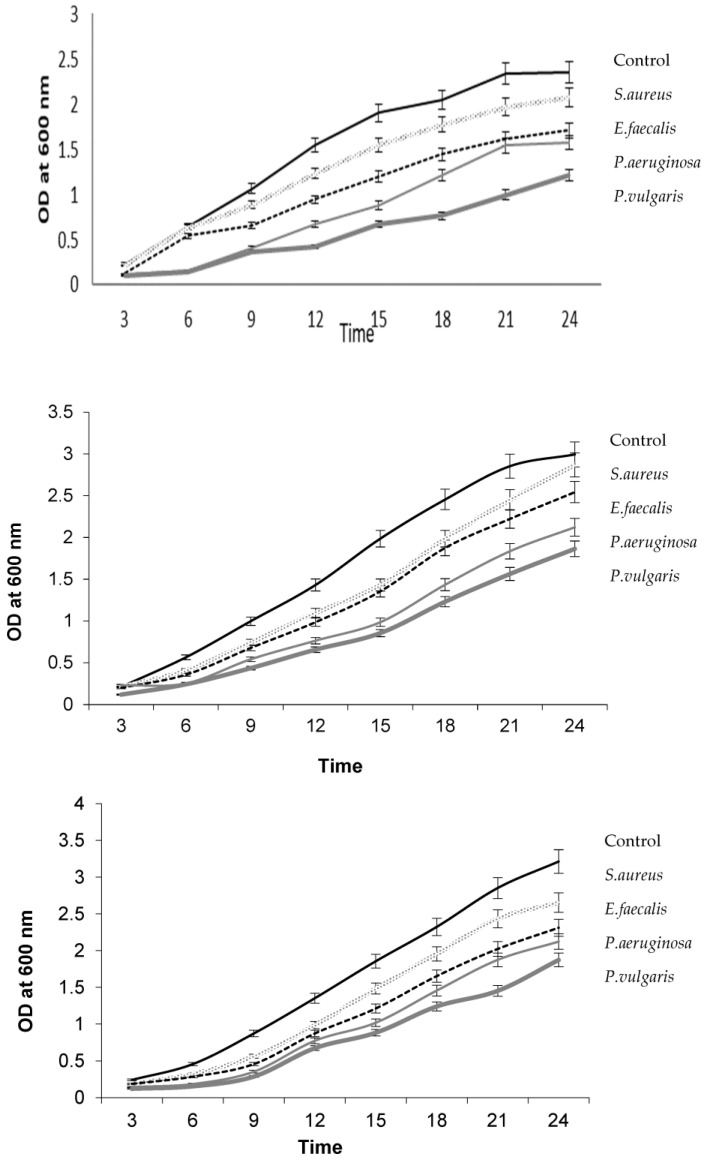
Growth-inhibitory activity of *H. pugilinus* hemocyanin against Gram-positive and Gram-negative bacteria (mean ± S.D, *n* = 3).

**Figure 3:**
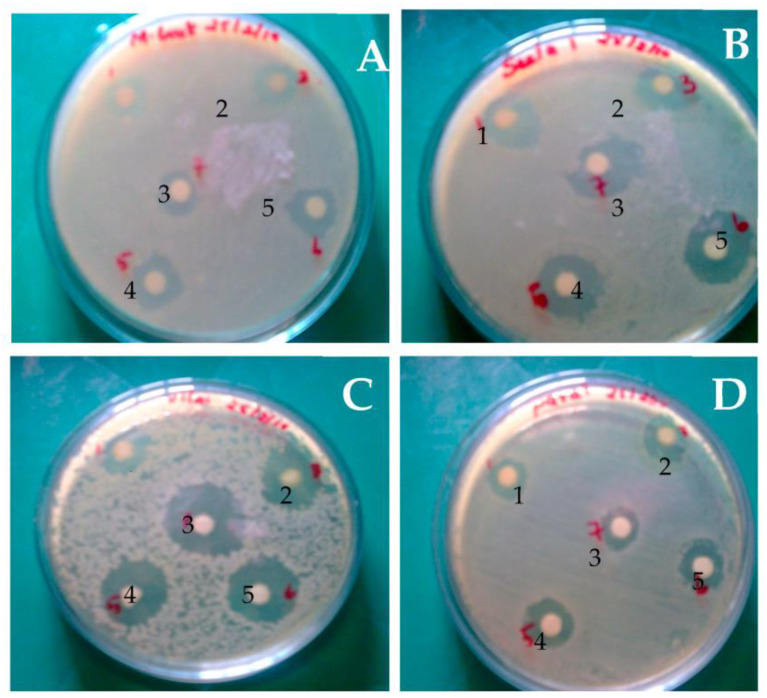
Antibacterial activity of *H. pugilinus* hemocyanin against Gram-positive *S. aureus* (**A**) and *E. faecalis* (**B**) and Gram-negative *P. aeruginosa* (**C**) and *P. vulgaris* (**D**).

*H. pugilinus* hemocyanin was purified via Sephadex G-100 gel filtration chromatography, and a single band with a molecular weight of 80 kDa was observed in SDS-PAGE (Figure 1, Supplementary Figure S1) with a retention time of 4.3 min. CD scanning showed a broad n-π transition centered around 210 nm and an intense p-p transition at 190 nm due to weak peptide bonds and β sheets. The sharp negative peaks near 208 nm and 220 nm represent the α-helix and β-sheets in the protein structure. In addition, the negative peak near 240 nm indicates an abundance of β-turn in the protein structure. Thus, the CD analysis revealed the presence of α-helix (4.5%), β-sheets (23.6%), β-turn (17.2%) and random coils (54.8%) in the secondary structure (Supplementary Figure S2). The ani-bacterial activity of *H. pugilinus* hemocyanin was evaluated against Gram-positive *Staphylococcus aureus* and *Enterococcus faecalis*, and Gram-negative *Pseudomonas aeruginosa* and *Pseudomonas vulgaris*. Bacterial growth is shown in the graphical growth diagram. There was *H. pugilinus* hemocyanin concentration-dependent inhibition of bacterial growth. The minimum inhibitory concentration (MIC) of *H. pugilinus* hemocyanin was found to be 1–5 μg/mL, at which the growth of both Gram-negative and Gram-positive bacteria was inhibited significantly (Figure 2 and Figure 3).

Invertebrate hemolymph hemocyanin is a multifunctional immune molecule that plays important roles in molting regulation, oxygen transport, the antigen immune response [38] and PO activity, and acts as a clotting factor, as a serine protease and as an antibacterial peptide [39]. In a few investigated crustaceans, including the Dungeness crab (*Cancer magister*) [40], Atlantic horseshoe crab (*Limulus polyphemus*), horseshoe crab (*Tachypleus tridentatus*) [34], kuruma prawn (*Penaeus japonicus*) [41], whiteleg shrimp (*Penaeus vannamei)* [39], deep-sea crustacean (*Bathynomus giganteus*) [42], crayfish (*Cherax quadricarinatus*) [35] and freshwater crayfish (*Pacifastacus leniusculus*) [29], hemocyanin’s conversion into a PO-like enzyme has been shown. Adachi et al. [41] reported that in *P. japonicas*, trypsin and chymotrypsin do not have the ability to convert Hc to HcPO. Antibacterial activity of hemocyanin from red swamp crayfish (*Procambarus clarkii*) has been reported [13]. Studies are still emerging and being reported on these novel antiviral and antibacterial compounds from marine molluscs due to their abundance and economic importance [43].

**Figure 4:**
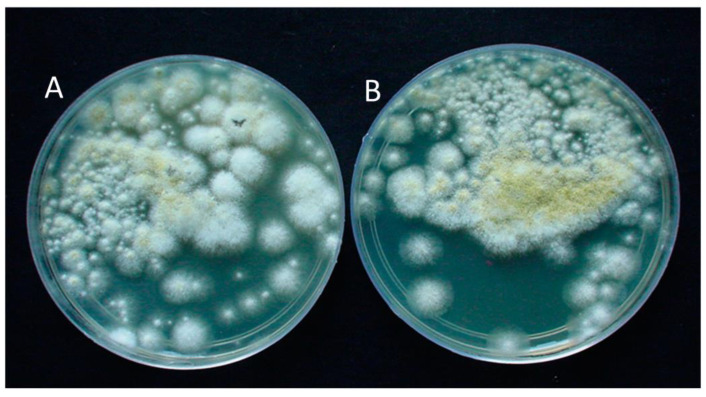
Antifungal activity of *H. pugilinus* hemocyanin against *A. niger* on plates. (**A**) Control and (**B**) hemocyanin-treated.

The first step in the initial attachment of biofilm-forming bacterial cells requires a substrate. Herein, the behavior of bacterial biofilm (Gram-positive and Gram-negative) attachment was analyzed using *H. pugilinus* hemocyanin. This molecule showed a potential inhibitory effect on microbial cells by restricting bacterial colony formation. This suggests that the onset of the biofilm’s adhesion to the matrix may have been inhibited at the initial stage itself. One of the key factors in the development of biofilms is bacterial adherence to hydrocarbons. Regarding the hydrophobicity index, effective bacterial biofilm inhibition was shown at a concentration of 40 μg/mL (the initial attachment of the bacterial strains on the surface). Supplementary Figure S3 illustrates the hydrophobicity index, which is partially reduced on the Gram-negative bacterial biofilms *P. aeruginosa* and *P. vulgaris*, whereas in the Gram-positive bacterial biofilms *S. aureus* and *E. faecalis*, the hydrophobicity index is significantly reduced. The microscopic analysis revealed that *H. pugilinus* hemocyanin is capable of inhibiting the biofilm growth of all four species, *S. aureus*, *E. faecalis*, *P. aeruginosa* and *P. vulgaris* (Supplementary Figure S4). The spectroscopic analysis of extrapolysaccharide (EPS) extracted from the control and *H. pugilinus* hemocyanin-treated samples revealed a significant reduction in EPS synthesis. The antifungal activity of *H. pugilinus* hemocyanin was tested on *C. albicans* and *A. niger*, wherein noticeable inhibition was observed on the *A. niger* plates (Figure 4).

**Figure 5:**
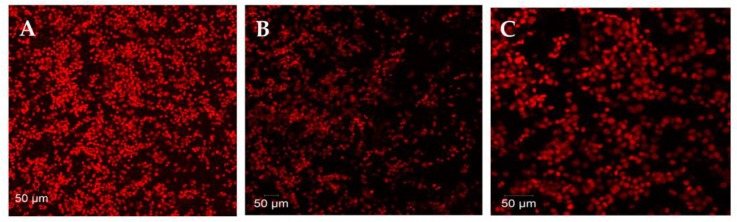
Confocal images of cytotoxicity of *H. pugilinus* hemocyanin to human colon adenocarcinoma cells at 2 different magnifications. (Control (**A**) *H. pugilinus* hemocyanin treated with cell lines (**B**,**C**)).

The biofilm formation was monitored via microtiter plate (MTP) assay using crystal violet dye and measured spectrophotometrically. By observing the biofilm architectural image under a light microscope (Supplementary Figure S4) and using CLSM-purified Hc (10–40 g/mL) after staining with CV and acridine orange, respectively, the percentage of biofilm biomass was determined (Supplementary Figure S5). When treated with purified Hc, bacterial biofilm formation was inhibited, contrary to the control glass pieces, which showed well-evolved biofilm growth of the tested bacteria. Treatment with purified Hc caused biofilm-forming bacteria to exhibit a collapsed biofilm architecture, suggesting effective biofilm inhibition. Using a staining technique that distinguishes between live and dead cells, the viability of human colon adenocarcinoma cells treated with *H. pugilinus* hemocyanin was based primarily on membrane integrity (Figure 5).

Molluscan hemocyanin possesses antibacterial, antiviral, antifungal and anticancer properties [8,19], and they are all intertwined with antimicrobial peptides [16]. Furthermore, it was proven that certain peptides and proteins from the hemolymph of molluscs and arthropods exhibit a wide range of antimicrobial activity against both Gram-positive and Gram-negative bacteria, as well as yeast [44,45]. Similar to this, pharmacologically active peptides have been identified in the hemolymph of the marine snail *R. venosa* and the garden snail *Helix lucorum* [46,47]. Mucus-derived medicines can be employed in a wide range of therapies, according to research by Dang et al. [48,49,50]. Dolashka et al. [51] conducted in vitro tests to compare the antitumor activity of hemocyanin molecules of *H. lucorum*, *R. venosa* and *H. aspersa*.

## 3. Materials and Methods

### 3.1. Animals

Fresh *H. pugilinus* with a size distribution of 4.96 cm were collected alive from the low-tidal coastal region of Pampan, Ramanathapuram, Tamil Nadu, India. They were then transported to the Biotechnology and Biosignal Transduction Lab and acclimated to laboratory conditions, a temperature of 28–30 °C and a photoperiod of 5–7 days, in an aerated space. The hemolymph was obtained by perforating the shell. Hemolymph separation and handling were performed at 4 °C to avoid cell aggregation [31].

### 3.2. Purification of H. pugilinus Hemocyanin

The hemocyanin was purified from the hemolymph according to Sivakamavalli et al. [52] with slight modifications. Briefly, a Sephadex G-100 gel filtration column (Bio-Rad, Hercules, CA, USA) was used, where hemolymph was equilibrated with CaCl_2_/TBS, stirred gently at 24 °C for 1–2 h, and applied to the Sephadex G-100 gel filtration column. Fractions of hemocyanin (Hc) were collected in 2 mL Eppendorf tubes at a flow rate of 5 mL h-absorbance detected at 340 nm, corresponding to copper-binding protein. Subsequently, SDS-PAGE (12% separating gel and 4% stocking gel) was performed to determine the molecular weight of the purified Hc [53]. The gel was stained with PAS staining solution and Coomassie brilliant blue for about 3 h. Following destaining, the gel was visualized under a gel documentation system (Bio-Rad) for clear bands. The purified Hc was examined using an HPLC C18 column (7.8 mm × 30 cm) using a linear gradient between 0.052% trifluoroacetic acid in 80% acetonitrile and 0.05% trifluoroacetic acid in water.

### 3.3. Minimum Inhibitory Concentration

The antimicrobial activity of Hc against Gram-positive *E. faecalis* (MTCC: 2729) and *Staphylococcus aureus* (MTCC: 9542), Gram-negative *Proteus vulgaris* (MTCC: 426) and *Pseudomonas aeruginosa* (MTCC: 4673), and the fungi *Candida albicans* (MTCC: 7315) and *Aspergillus niger* (MTCC: 2208) was assessed. To determine the minimum inhibitory concentration (MIC) of the hemocyanin, a broth dilution method of crude extract of *H. pugilinus* was performed. Serially diluted *H. pugilinus* extract at different concentrations was added into the wells. A total of 10 μL of test micro-organism suspension was added to 100 μL of nutrient broth. To top up the well, sterile water was added up to the 20 μL mark. The plates were incubated at 37 °C for 24 h, followed by the addition of 3-(4,5-dimethylthiazol-2-yl)-2,5-diphenyl tetrazolium bromide (MTT) to each well. A change in color to violet was an indicator of microbial development. When the color of the well remained the same, this indicated inhibition of the development of microbes.

Using the disc well diffusion method, *H. pugilinus* hemocyanin fractions were tested for potential antimicrobial activity against both Gram-positive and Gram-negative bacteria. The growth of Gram-positive *S. aureus* and *E. faecalis* and Gram-negative *P. aeruginosa* and *P. vulgaris* was used to determine the antimicrobial efficacy of *H. pugilinus* hemocyanin. The test organisms were injected into Luria Bertani broth (pH 7.4), where they remained for 8 h. On Luria Bertani agar plates, isolates were dispersed using sterilized cotton swabs. Wells (7 mm diameter) were bored into the surface of the agar using a sterilized gel borer. One hundred microliters of sterilized distilled water (negative control) and different concentrations of emocyanin, ranging from 2 μg to10 μg/mL, were added to separate wells. A standard antibiotic disc was placed on the agar surface as a positive control, and then the plates were incubated at 37 °C for 24 h.

### 3.4. Antibiofilm Assay

*H. pugilinus* hemocyanin biofilm inhibition was studied in *E. faecalis, S. aureus, P. vulgaris* and *P. aeruginosa* bacterial colonies (1 × 10^6^ CFU mL^−1^) grown on glass pieces (diameter 1 × 1 cm), and the glass pieces were placed in polystyrene 24-well plates, and 1 mL of nutrient broth augmented with diverse concentrations ranging from 10–40 µg/mL of *H. pugilinus* hemocyanin each were poured, and then, incubated for 24 h at 37 °C. The glass pieces were stained with 0.04% crystal violet. The excess stain was washed off using PBS and the stained glass pieces were examined under a Nikon inverted research microscope (ECLIPSE Ti100,Bahadurgarh, Haryana, India) at 40× magnification. Subsequently, one more set of glass pieces, with biofilms grown as above, were washed with PBS and stained with acridine orange (0.1%). Later, the biofilm growth was quantified using a confocal laser scanning microscope (CLSM-Carl Zeiss LSM 710) running Zen 2009 software (Carl Zeiss, Germany) with a 488 nm argon laser and a band path of 500–640, along with a band pass emission filter.

### 3.5. Antifungal Activity

Using the agar well diffusion method on Potato Dextrose Agar (PDA) medium, the antifungal activity of the *H. pugilinus* hemocyanin was examined. On solid plates, the inocula were spread using fungal suspension-moistened sterile swabs. The antifungal activity of the *H. pugilinus* hemocyanin was evaluated using the methodologies proposed. *H. pugilinus* hemocyanin present in issue slices with various concentrations (5–25 μg) of *H. pugilinus* hemocyanin/mL were used in the sterilized PDA. Only PDA was evident in the control dishes. Each Petri dish containing *C. albicans* and *A. niger* mycelium was placed in the center and incubated at 27 ± 1 °C. At intervals of 24 h, the colony’s diameter (cm) was measured. When the mycelia of the fungi grew to the edges of the control dish, the experiment was considered to be over. At the conclusion of the experiment, the antifungal index (AI) was derived using the formula below:

AI (%) = (1 − D1/D2) × 100(1)

In which D1 represents the colony diameter of the test dishes, whereas D2 represents the colony diameter of the control dish.

### 3.6. Statistical Analysis

Each analysis was performed in triplicate and the data are reported as the mean ± standard deviation (SD). One-way ANOVA was used to analyze significant differences. Differences at *p* < 0.05 were deemed statistically significant.

### 3.7. Cell Cytotoxicity

The cells (HCT 15—human colon adenocarcinoma cells) were seeded at 1 × 10^5^ cells/well in 24-well plates. After overnight incubation, the medium was replaced with a maintenance medium (DMEM) without FBS and containing varying concentrations of *H. pugilinus* hemocyanin, and incubated for 48 h. Later the plates were examined microscopically for any sort of cytotoxicity. The cells were washed with PBS, fixed in methanol:acetic acid (3:1 *v*/*v*) for 10 min and stained with 50 lg/mL of propidium iodide for 20 min. The nuclear morphology of apoptotic cells with condensed/fragmented nuclei was examined under a Carl Zeiss Confocal microscope (LSM 710).

## 4. Conclusions

Invertebrates are a major source of immune molecules (macromolecular proteins), natural small molecules and bioactive compounds that work against microbial pathogens. There is a compelling need for the development of pharmaceuticals in the aquaculture industry through marine biotechnology to enhance the aquaculture/blue economy. In this study, the vital antimicrobial protein hemocyanin of the snail *H. pugilinus* was separated, identified and isolated, and its bioactivity was tested, which highlights the potential of this commercially important marine mollusc to be used for therapeutic applications. Though much more comprehensive investigations are needed to advance research on this important compound from laboratory scale to clinical trials, it is obvious that such molecules isolated from snails could be used as a potential avenue to novel antibacterial drug discovery. Our results show that hemocyanin from the marine snail *H. pugilinus* possesses strong antibacterial and antibiofilm activities against both Gram-positive and Gram-negative bacteria. Considering our antibacterial, antibiofilm and cytotoxicity results, these molecules highlight the effectiveness of the immune system of these marine animals. To further understand the chemical makeup of the specific molecules, extensive research aimed at isolating these bioactive compounds should be the next step.

## Data Availability

All data are available in this manuscript.

## Supplementary Materials

The following supporting information can be downloaded at: https://www.mdpi.com/article/10.3390/ijms241411494/s1.
