# Supplementary material for: Exploring the Antimicrobial Potential and Biofilm Inhibitory Properties of Hemocyanin from Hemifusus pugilinus (Born, 1778)
Source: Int J Mol Sci. 2023 Jul 15;24(14):11494. doi: 10.3390/ijms241411494 (PMC10380319; doi:10.3390/ijms241411494)
Supplement: Supplementary file 1 [file ijms-24-11494-s001.zip › ijms-2457147-supplementary.pdf]

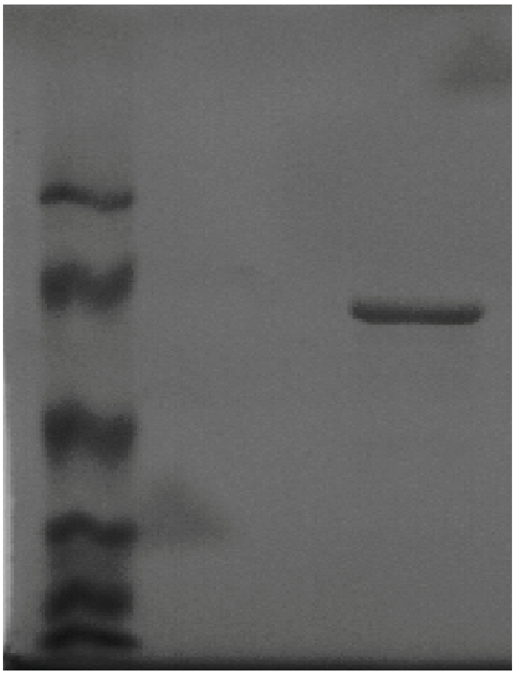

Supplementary Figure S1. Silver staining of *H. pugilinus* hemocyanin.

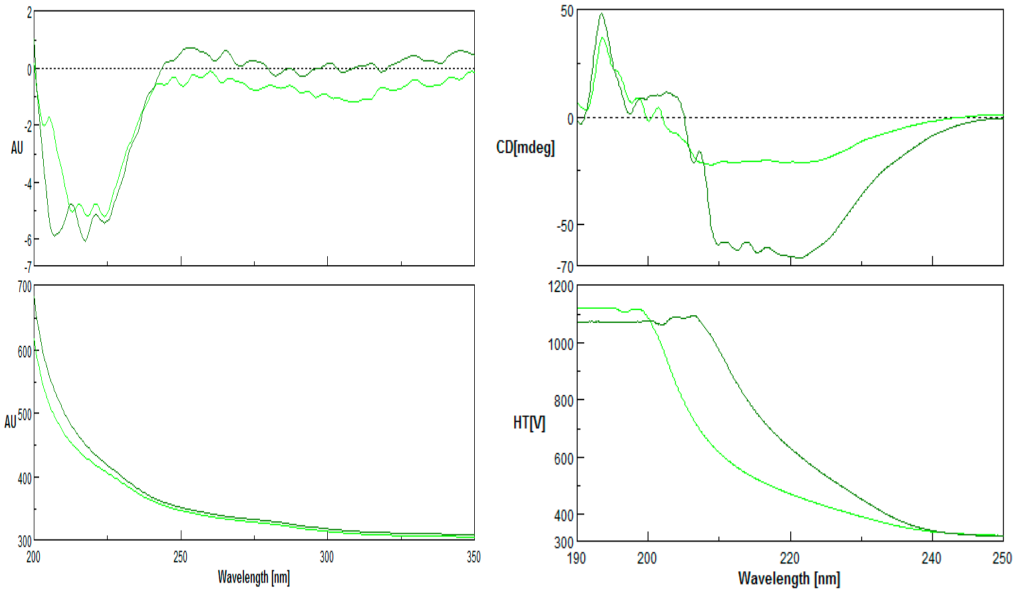

(**a**) α-Helices (**b**) β-Sheets

Supplementary Figure S2. Circular dichroism spectrum of *H. pugilinus* hemocyanin.

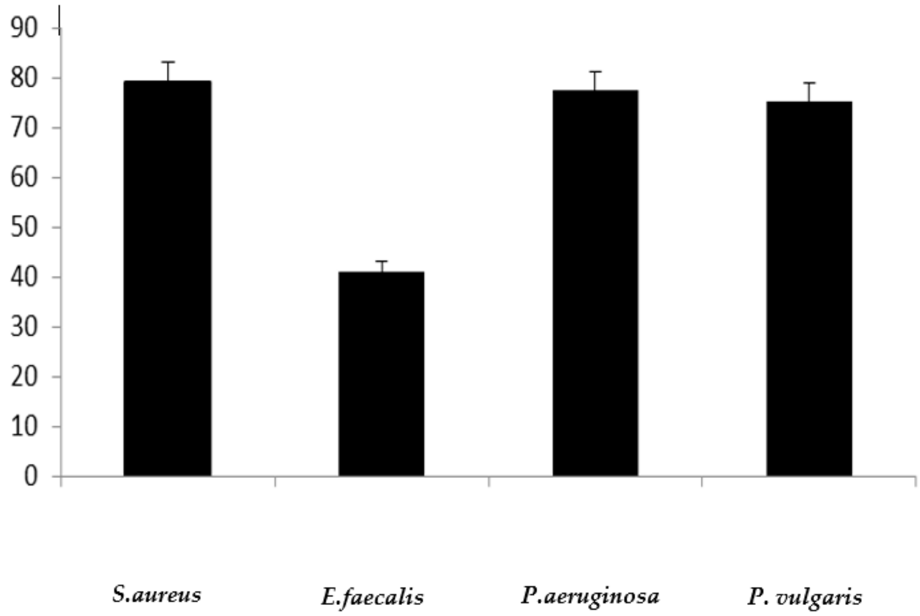

Supplementary Figure S3A. Analysis of biofilm formation through initial attachment assay, which showed the percentage of biofilm inhibition by *H. pugilinus* hemocyanin.

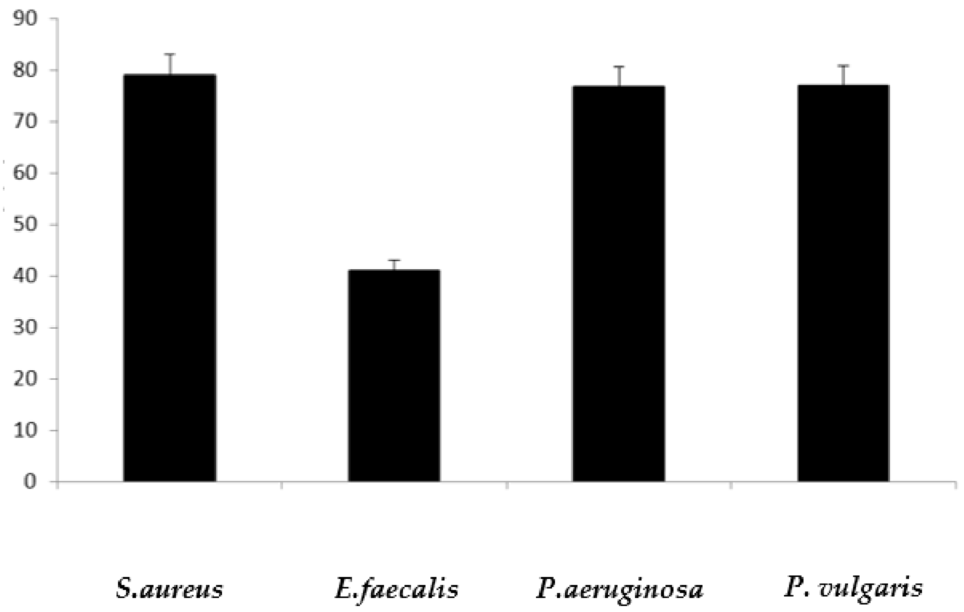

Supplementary Figure S3B. Percentage EPS production in Gram-positive and Gram-negative bacteria treated with *H. pugilinus* hemocyanin.

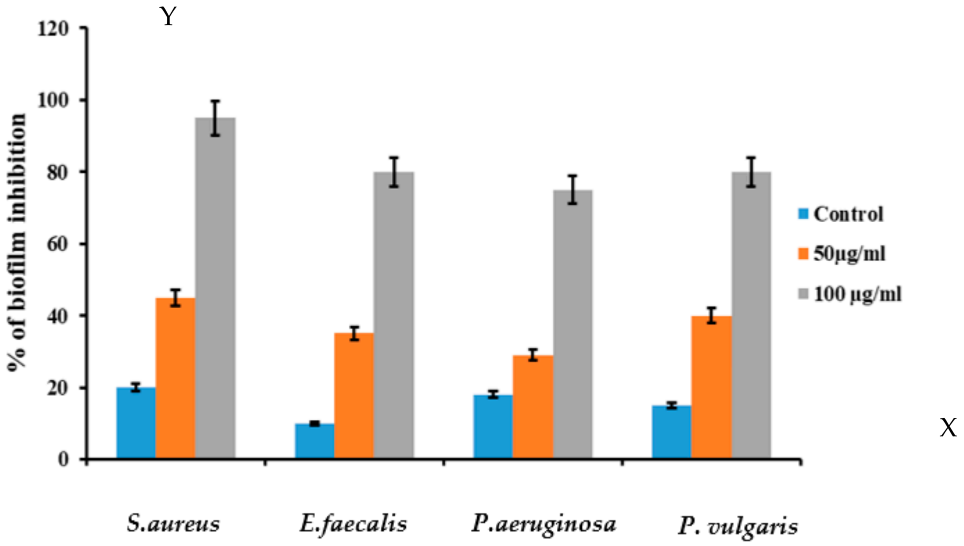

Supplementary Figure S3C. Biofilm inhibitory concentration of *H. pugilinus* hemocyanin. Bars with different letters indicate the significant differences between treatments (ANOVA, Tukey's HSD test, p<0.05).

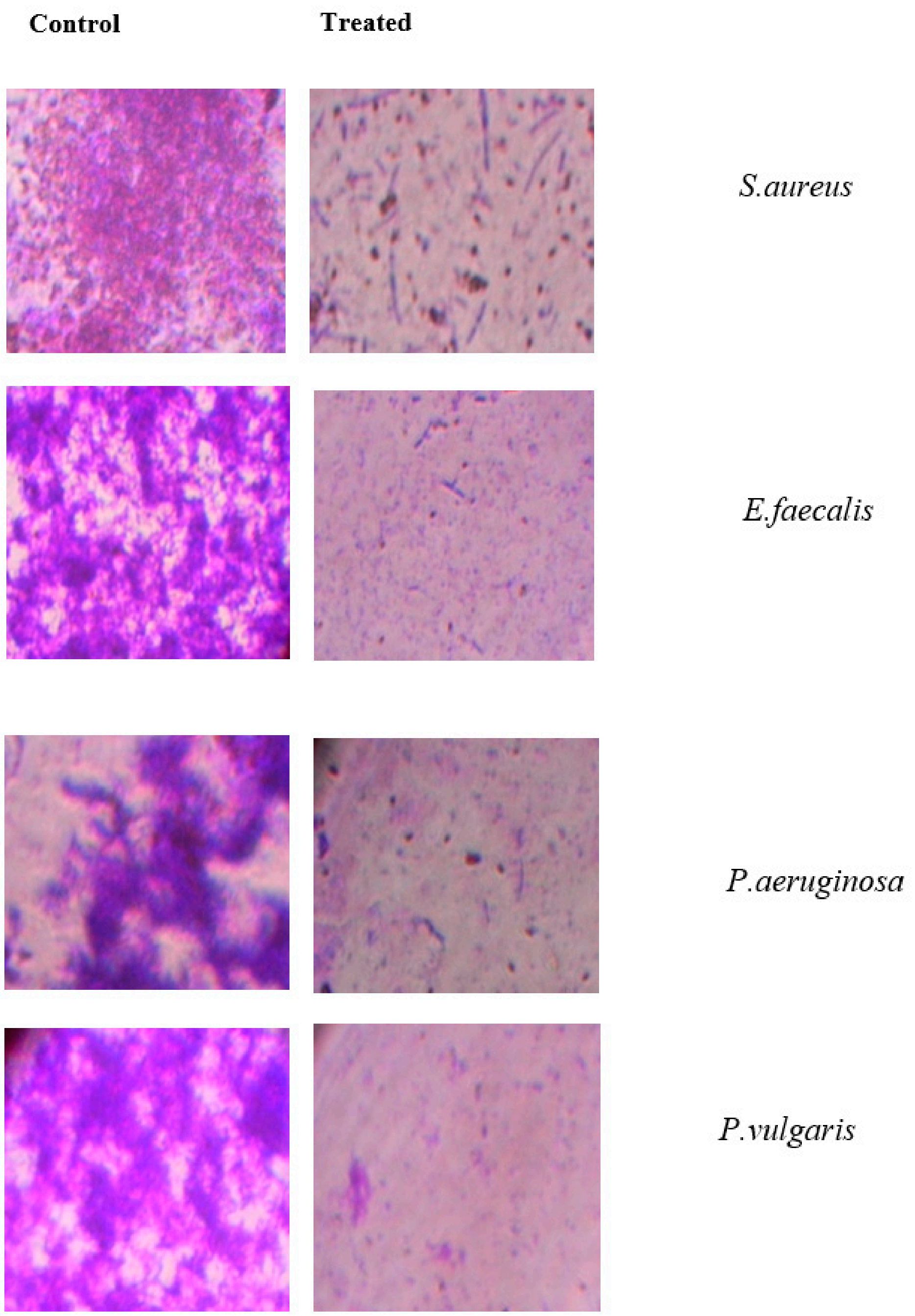

Supplementary Figure S4. Light microscopic images of biofilm growth of Gram-positive and Gram-negative bacteria (Left panel, control; right panel, *H. pugilinus* hemocyanin treated).

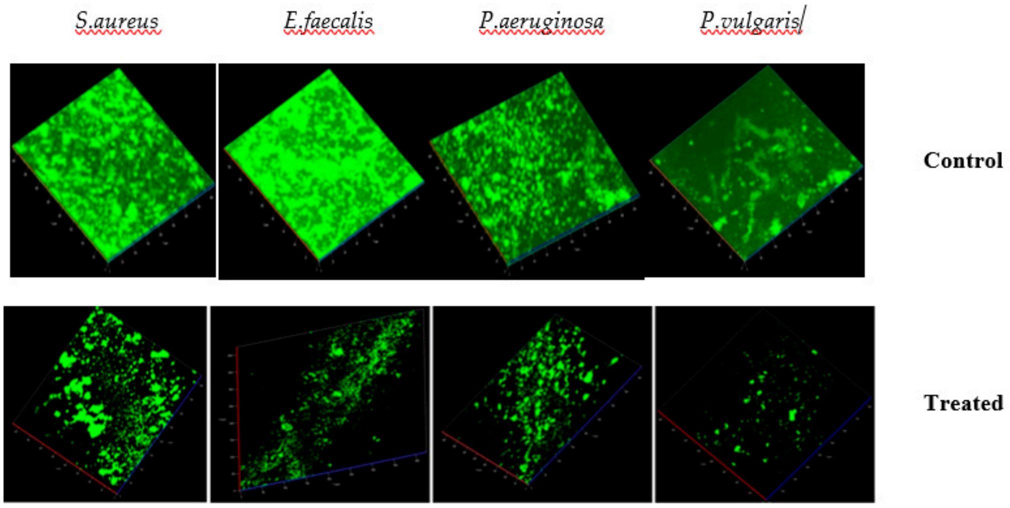

Supplementary Figure S5. Confocal Laser Scanning Microscopy (CLSM) images of biofilm growth of Gram-positive and Gram-negative bacteria (Top row, control; bottom row, *H. pugilinus* hemocyanin treated) (2D images).
